# Personalized ESM monitoring and feedback to support psychological treatment for depression: a pragmatic randomized controlled trial (*Therap-i*)

**DOI:** 10.1186/s12888-021-03123-3

**Published:** 2021-03-10

**Authors:** H. Riese, L. von Klipstein, R. A. Schoevers, D. C. van der Veen, M. N. Servaas

**Affiliations:** Interdisciplinary Center Psychopathology and Emotion regulation, Department of Psychiatry, Universitair Medisch Centrum Groningen, University of Groningen, P.O. Box 30.001 (CC72), 9700 RB Groningen, The Netherlands

**Keywords:** Depression, Ecological momentary assessment (EMA), Mental health care, Personalized experience sampling method (ESM) monitoring and feedback, Pragmatic randomized controlled trial

## Abstract

**Background:**

Major depressive disorder (MDD) is a highly prevalent mental disorder with large disease burden, high levels of relapse or persistence, and overall suboptimal outcomes of protocolized pharmacological and psychotherapeutic treatments. There is an urgent need to improve treatment effectiveness, possibly through systematic treatment personalization. In psychotherapeutic treatments this can be achieved by case conceptualization. To support this process, we developed the Therap-i module, which consists of personalized Experienced Sampling Methodology (ESM) and feedback. The Therap-i module is integrated into outpatient psychotherapeutic treatment as usual (TAU) for depression. The study aim is to investigate the efficacy of the Therap-i module in decreasing symptomatology in unresponsive or relapsing patients diagnosed with MDD. We hypothesize that the Therap-i module will contribute to TAU by i) decreasing depressive symptoms, and ii) improving general functioning, therapeutic working alliance, and illness perception. This paper provides details of the study rationale, aims, procedures, and a discussion on potential pitfalls and promises of the module.

**Methods:**

Patients diagnosed with MDD (*n* = 100) will enrol in a pragmatic two-armed randomized controlled trial. Randomization is stratified according to the patient’s treatment resistance level assessed with the Dutch Method for quantification of Treatment Resistance in Depression (DM-TRD). All fill-out the Inventory of Depressive Symptomatology Self Report (IDS-SR), Outcome Questionnaire (OQ-45), Illness Perception Questionnaire Mental Health (IPQ-MH), and Work Alliance Inventory Self Report (WAI-SR). In the intervention arm, through close collaboration between patient, clinician, and researcher, a personalized ESM diary is developed based on the patient’s case conceptualization. During the ESM monitoring period (8 weeks, 5 assessments/day), patients receive feedback three times, which is discussed among the abovementioned three parties. Both patients and clinicians will evaluate the Therap-i module.

**Results:**

Data collection is ongoing.

**Discussion:**

This is the first study in which personalized ESM and feedback is integrated in outpatient psychotherapeutic TAU for depression. The labour intensive procedure and methodological pitfalls are anticipated challenges and were taken into account when designing the study. When hypotheses are confirmed, the Therap-i module may advance treatment for depression by providing insights into personalized patterns driving or perpetuating depressive complaints.

**Trial registration:**

Trial NL7190 (NTR7381), registered prospectively 03-08-2018.

**Supplementary Information:**

The online version contains supplementary material available at 10.1186/s12888-021-03123-3.

## Background

Major depressive disorder (MDD) is a highly prevalent mental disorder with large disease burden for patients, their relatives, and society [1, 2]. Due to its intermittent course, where remission is often followed by relapse [3] or persistence of depressive complaints, there is a large and growing need to improve treatment outcome. This is particularly the case for those who have been unresponsive, or insufficiently responsive, to stepwise protocolized pharmacological and psychotherapeutic treatments [4]. These patients are considered difficult-to-treat and further personalization of treatment is needed. Clinicians typically personalize psychological treatment through (re-)investment in a patientۥ s case conceptualization [5]. In case conceptualization patients and clinicians develop a holistic working theory about the patient’s psychopathology.

The core feature of case conceptualization is the active participation of both the patient and clinician. In close collaboration, both combine their unique expertise to describe and explain the patient’s psychopathology in a holistic theory, or case concept. In short, case conceptualization involves: i) describing the presenting patient’s psychopathology, ii) providing a working hypothesis about the psychological mechanisms that drive or maintain a patient’s psychopathology, iii) organizing and integrating patient information based on the judgment of the clinician, and iv) informing diagnosis and treatment [5]. However, although well-established, there is no gold standard for the methodology and theoretical basis of case conceptualization [5–7]. This results in a lack of consensus on the essential features of case conceptualization and low inter-rater reliability between case concepts of different clinicians [8, 9]. Contributing factors might be that a case concept is based on retrospective information and depends on the questions asked by a particular clinician. These issues can be addressed by systematic momentary monitoring in a patient’s normal daily-life and subsequently providing feedback on this information during a subsequent regular consult. A scientific method to obtain this goal is the experienced sampling method (ESM).

ESM involves repeated sampling of momentary affect, cognitions, and/or behavior, typically via a patient’s own smartphone [10]. Prolonged ESM monitoring (i.e., weeks, months) results in time-series data, which can reveal the dynamic course of psychopathology in individuals [11, 12]. ESM holds the promise to advance the case conceptualization process for notable reasons: i) ESM provides patients and clinicians access to momentary information, thereby reducing retrospective bias [10, 13, 14]; ii) ESM promotes the patients’ reflections and insights in his/her own psychopathology by intensive self-monitoring [15]; and iii) ESM derived time-series data can be summarized into personalized feedback reports with intuitive visualizations of the course of the data (e.g., [16]). Such feedback may be further improved through more sophisticated analyses on the time-series data. A method that may hold particular promise for case conceptualization are person-specific network models [17, 18]. Proof-of-principle studies showed that results from such network models may be used as a starting point for a dialog between the patient and clinician during regular consults (e.g. [16, 19, 20].

The efficacy of ESM-based feedback as a therapeutic tool in decreasing depressive symptoms in outpatients diagnosed with depression was first shown in a pioneer randomized controlled trial (RCT, *n* = 102 [21];). The treatment as usual (TAU) condition primarily consisted of pharmacotherapy, with less than 10% of the patients receiving additional psychotherapeutic treatment. In the experimental condition of this study, patients received TAU and monitored their momentary positive affect for 6 weeks. Every week a feedback report was generated from the collected time-series data by a clinical researcher, who discussed the feedback with the patient and his/her clinician. In another, more recent pragmatic RCT in patients indicated for treatment for depression (*n* = 161, [22, 23]), efficacy of ESM-based feedback added to pharmacological and/or psychotherapeutic TAU was not established. In this study, patients monitored their momentary positive and negative affect for 4 weeks. ESM monitoring started after the intake procedure while the patient was waiting for psychotherapeutic treatment, continued monitoring during treatment and weekly feedback was emailed to the patient. The fourth feedback report was discussed with the patient and a research assistant. The authors concluded that, while ESM-based feedback in depression treatment was highly appreciated by both patients and clinicians in their study, its promise to augment the efficacy of regular depression treatment was not met. However, they cannot rule out that it advanced other domains (e.g. acquire better self-insight), or provided a more efficient way of delivering care (i.e. more patients may be treated when combining face-to-face sessions with ESM compared to full face-to-face treatment). Besides notable differences between the two ESM studies outlined above [21, 23], they have in common that ESM was neither personalized nor fully integrated into patient’s psychotherapeutic treatment.

Interestingly, recent research findings from patients and clinicians stress the importance of personalizing ESM assessment and feedback [15]. Furthermore, Bos and colleagues recommended that ESM should be implemented by an interdisciplinary team of patients, clinicians, researchers, and information technology specialists. These recommendations are in line with the clinical process of case conceptualization outlined above. The present study follows these recommendations by implementing them in a new ESM-based intervention: the Therap-i module. In Therap-i fixed ESM items will be supplemented with personalized items to cover core elements of the case concept of each individual. Patients and their clinicians will collaborate, together with the researcher, in personalizing ESM and, discussing ESM-derived feedback results in feedback sessions to advancing the case conceptualization process. Therap-i feedback includes graphs on daily fluctuations of scores on the ESM items, their associations, and contextual information (e.g. notes on (un) pleasurable events, company and activities). Researchers will assist patients and their clinicians during the entire procedure. This approach may anchor the case concepts more robustly in the patient’s narrative during psychotherapeutic treatment.

### Study aims

We aim to test the efficacy of the Therap-i module as a supportive tool in psychotherapeutic TAU in MDD patients, who have been insufficiently responsive to protocolized treatments for depression. We hypothesize that the Therap-i module is effective in these patients in i) decreasing depressive symptom severity (primary outcome), ii) increasing general functioning, iii) increasing the therapeutic working alliance, and iv) improving illness perception (more specifically, increased illness insight, increased personal control over the illness, increased control through treatment, and reduces emotional representation of the illness). After treatment, self-management strategies will be examined and the Therap-i module will be evaluated by both patients and clinicians with quantitative and qualitative instruments. This paper provides a detailed overview of the patient inclusion procedure, instruments, and ESM protocol used in the Therap-i study. Furthermore, potential pitfalls and promises of the module are discussed.

## Methods

### Participants

**Fig. 1:**
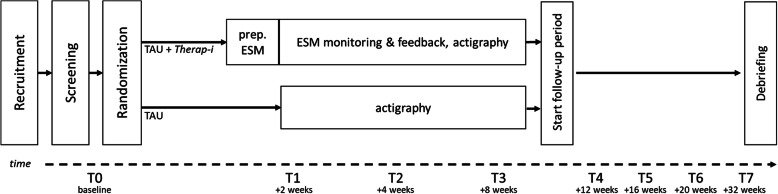
The *Therap-i* study design (upper part l) and the scheduled questionnaire assessments (lower part). Details are given in the method section. Note: TAU, treatment as usual; prep. ESM, preparation experience sampling method

The Therap-i study is a pragmatic RCT to evaluate the efficacy of the Therap-i module as a tool to support psychological TAU in patients diagnosed with MDD. In the RCT a control group, which receives TAU, is compared with an experimental group, which receives ESM-supported TAU (i.e. the Therap-i module). The design of the Therap-i study is shown in Fig. 1. The trial was registered prospectively on August 3th in 2018 in the Dutch Trial Register (NTR7381, www.trailregister.nl). The study was approved by the Medical Ethical Committee of the University Medical Center Groningen (UMCG, METc2018/434). All patients provide written informed consent prior to participation. According to UMCG policies for studies with negligible risk, an UMCG study monitor was appointed and a monitoring plan was written to assure quality of research. All investigators working on the study have received training and are certified in study conduct, informed consent, and risk assessment (BROK® certified).

The in- and exclusion criteria are given in Table 1. We aim to recruit 100 outpatients diagnosed with MDD, who have been unresponsive, or insufficiently responsive, to current or past protocolized psychotherapeutic treatments. Patients are recruited from multiple mental health care institutions in the North of the Netherlands. Patients are eligible for participation if they are starting with outpatient psychotherapeutic treatment, or if they are currently in treatment and their clinician decides to re-invest in case conceptualization and to personalize treatment. No limitations are applied regarding TAU, although use of medication and psychological interventions will be systematically monitored. Patients are informed that participation is voluntary and that not participating will not affect their TAU. Patients will be rewarded with €30 for their participation.

**Table 1:** Eligibility criteria for enrolment in the *Therap-i* study

Inclusion criteria	Exclusion criteria
- Meets a MDD diagnosis according to the Diagnostic Statistical Manual (DSM-V) within the past six month. - Has received a primary protocolled psychological intervention for depression, consisting of four months of (cognitive) behavioural therapy, interpersonal therapy or brief psychodynamic therapy, and which is evaluated as not effective (enough) to repeat or continue by the clinician responsible for the participant. - Receives, or is scheduled to receive, policlinic psychological treatment, with weekly one-on-one consults with a clinician for at least 8 weeks. - Aged between 18 and 65 years.	- Meets a diagnosis of MDD with psychotic features, bipolar disorder, substance use disorder, schizophrenia spectrum and other psychotic disorder, neurocognitive disorder in the past six months. - Has a history of treatment with electroconvulsive therapy. - Insufficient mastery of the Dutch language. - Inability or unwillingness to use a smartphone.

### Procedure

#### Recruitment

Potential participants receive a brochure and a contact consent form from their treating or directing clinician. After consent for contact, researchers contact the patient to provide further information about the study. Eligibility also includes approval of the treating clinician in case a patient is contacted via a directing clinician. After 1 week to consider participation, the patient is contacted again to inquire about their willingness to participate in the study and if so, to conduct a short screening for study eligibility. Research Electronic Data Capture (REDCap) software [24, 25] is used to register all contacts with (potential) participants and to manage logistics of the research procedure. Details on data management are given below.

#### Screening

Prior to enrolment, patients are screened on psychiatric disorders that are listed in the in- and exclusion criteria with the Mini-Schedules for Clinical Assessment in Neuropsychiatry (mini-SCAN) and assessed on treatment resistance with the Dutch Method for quantification of Treatment Resistance in Depression (DM-TRD). The mini-SCAN is a validated semi-structured interview to assess psychiatric disorders [26]. Parts of the interview that are relevant to the diagnoses mentioned in the in- and exclusion criteria, are used to confirm their respective presence or absence. The DM-TRD is a questionnaire to assess the level of treatment resistance in depression [27], which is used for stratification in the study (further details given below).

#### Randomisation and stratification

Randomization is outsourced to the research support office of the UMCG (Data Management & Research IT) and programmed in ALEA FormsVision (www.aleaclinical.eu). Participants are randomly allocated to the experimental or control group (allocation ratio 1:1). Randomization is stratified according to the level of treatment resistance based on the DM-TRD (cut-off score >11, [27]). A random block size will be used with a minimum of 4 and a maximum of 8, resulting in blocks of 4, 6 or 8. After *n* = 25, it is evaluated whether the chosen cut-off for stratification is effective. An adjustment of the cut-off will be considered if the chosen stratification cut-off fails in filling the cells in the randomization blocks. Failure is defined as one stratum containing ≥75% of the included participants. If this is the case, the stratification cut-off score will be replaced with the average DM-TRD score calculated on the participants included until then. This evaluation process is an iterative process, which will be repeated after every *n* = 25 inclusions. Allocation concealment is guaranteed through the use of ALEA FormsVision, making the randomisation sequence unpredictable for investigators. The ultimate goal of this process is to equally distribute the level of treatment resistance of the included patients across the two study groups.

### The Therap-i module

For participants in the experimental group, the Therap-i module is added as a supportive tool to their psychotherapeutic TAU. The Therap-i module involves; i) a resilience interview, ii) the development and initialisation of an ESM diary, iii) ESM monitoring, and iv) three feedback sessions.

First, in the resilience interview, we focus on assessing strengths of the patient. This aspect can be relatively underrepresented in clinical practice because of the primary focus on psychopathological complaints. Personal strengths and resilience factors are inquired by asking questions originating from solution-focused therapy [28]*,* such as “How would you describe yourself as a person before you got sick?”, “Can you describe one or several uplifts in the last month?”

Second, ESM items are developed for each patient individually. Items cover five domains that are important for case conceptualization: affect, behavior, cognition, body, and context. The ESM diary contains items that are assessed multiple times per day and items that are assessed once per day (morning/evening). It includes 22 fixed items (Table S1.1) and up to about ten personalized items. The fixed items are part of the ESM item bank developed in our department for the PErsonalized Treatment by Real-time Assessment (PETRA; www.petrapsy.nl [29];) project. The number of personalized items is flexible, but patients will be advised to limit the total number of items to 30 in order to manage measurement strain [15, 30]. The personalized items are developed in the ESM development session (45 min). Patient, clinician, and researcher collaborate to identify important personal aspects that trigger, perpetuate or influence psychiatric complaints to create the personalized ESM items. The researcher provides suggestions based on information from the electronic patient health record and the resilience interview. The researcher checks whether identified aspects are covered by items in the PETRA item bank. If this is not the case, a new item will be created based on a set of criteria for high-quality ESM items given in the Supplementary Materials S2. After the first and second feedback session (explained in detail below), it is possible to add diary items if new insights were obtained. The session in which the ESM diary is personalized is immediately followed by a 30-min instruction session between the patient and researcher, which also includes a practice run.

Third, patients fill out their personalized ESM diary 5 times a day for 8 weeks. The personalized ESM diary is initiated in a web-based flexible interface on our secured server system Routine Outcome and QUality Assessment (RoQua, [31]). Timing of assessments will be personalized to fit natural wake-sleep rhythm; the first daily assessment will be 2–3 h after waking-up in the morning. The system is programmed to send text messages (beeps) with links to the online questionnaires to the patient’s smartphone. This yields a maximum of 280 data points for the personalized feedback. Patients will be called 48 h after starting with ESM monitoring for motivation and to ask whether they have questions or experience technical problems. Every other day, the frequency of filling out ESM diaries is monitored in RoQua. When more than three subsequent assessments are missed, patients will be contacted to check whether they experience problems. Patients are instructed to complete the ESM diaries as soon as possible after receiving the beep, preferably within 10 min, and that they have limited time to respond. When no response is received, patients will be reminded after 10 min, and the link will be closed after 30 min. During the ESM monitoring period the patient can contact the researcher 24/7 when needed.

Fourth, patients receive personalized feedback during a regular consult with their clinician and the researcher after 2, 4, and 8 weeks of ESM monitoring. Dynamic visualizations of ESM data are provided with in-house developed software for personalized feedback (in R, using packages RMarkdown and Shiny by L. von Klipstein [32, 33];). In the feedback sessions the focus is on combining quantitative information from ESM rating scales with qualitative (contextual) information from patient-entered text during monitoring. In line with previous feedback report procedures [21, 23], the reports contain increasingly rich information across the treatment period. Patients receive feedback on raw course ESM time-series data after 2 weeks, and after 4 and 8 weeks monitoring additionally feedback on ESM time-series derived network models is available. Detailed information on the feedback module is beyond the scope of the current design paper and described elsewhere (von Klipstein et al., in preparation). A log of the graphs discussed along with notes of the session will be saved as a pdf file and sent to the patient and clinician for reference.

### Study parameters

#### Questionnaire assessments

Figure 1 shows the schedule of questionnaire assessments, and Table 2 provides the instruments used at each wave (T0-T7). All questionnaire were assessed in Dutch. All participants fill out questionnaires over a period of 8 months: before and during the first 8 weeks of their treatment period (T0-T3) and during a follow-up period (T4-T7). Questionnaire data are collected via our secured RoQua web application developed to monitor outcomes in health care and research [31]. Participants receive an email with a link to fill out the questionnaires. When necessary, participants receive a reminder to fill out the questionnaires by email after 2 and 4 days and a call by a research assistant after 4 days.

**Table 2:** Overview of instruments

		P	T0	T1	T2	T3	T4	T5	T6	T7
				ESM period						
**Screening/Stratification**										
Mini-SCAN	psychiatric diagnoses	X								
DM-TRD	treatment resistance	X								
**Baseline characteristics and others**										
Questionnaire	demographics, medication, prior treatment		X							
TAS-20	alexithymia		X							
LEIDS-RR	cognitive reactivity		X							
Long-term difficulties questionnaire	long-term difficulties		X							
DAS-A-17	dysfunctional attitudes		X							
CTQ-SF	childhood trauma		X							
Happiness index	happiness		X				X			
Brugha	life events						X			
Single item	medication switches						X			X
Single item (clinician)	type of psychological intervention					X				
**Outcomes**										
IDS-SR	depressive symptom		X	X	X	X	X	X	X	X
OQ-45	psychol. functioning		X	X	X	X	X	X	X	X
WAI-SR	therapeutic alliance^a^			X	X	X	X	X	X	X
IPQ-MH	illness perception		X	X	X	X	X	X	X	X
QSRD	self-management						X			
**Daily assessments**										
ESM^b^	momentary affect and other factors			−5 times a day-						
Actigraphy	physical activity			-continuous-						
**Evaluation Therap-i module**^b^										
Questionnaire	participant evaluation						X			
Questionnaire	clinician evaluation					X				
Interview	participant evaluation						X			

Note: P, pre-baseline; T0, baseline measurement; T1 till T7, follow-up waves (see method section for details); *CTQ-SF* Childhood Trauma Questionnaire Short Form, *DAS-A-17* Dysfunctional Attitude Scale form A, *DM-TRD* Dutch Method for quantification of Treatment Resistance in Depression, *ESM* Experienced Sampling Method, *IDS-SR* Inventory Depressive Symptomatology - Self-Report, *IPQ-MH* Illness Perception Questionnaire Mental Health, *LEIDS-RR* Leiden Index of Depression Sensitivity 2nd Revision, *Mini-SCAN* Mini-Schedules for Clinical Assessment in Neuropsychiatry, *OQ-45* Outcome Questionnaire-45, *QSRD* Questionnaire Self-management in Recovery of Depression, *TAS-20* Toronto Alexitymia Scale-20, *WAI-SR* Work Alliance Inventory Short form Revised. ^a^, WAI-SR is assessed at baseline if the patient already knows their clinician (see method section for details); ^b^ = experimental group only

Outcomes are assessed at all waves with the: i) Inventory of Depressive Symptomatology Self-Report (IDS-SR) to assess depression symptom severity [34–36]; ii) the Outcome Questionnaire (OQ-45) to assess changes in psychosocial functioning within three domains, including symptom distress, interpersonal functioning and social role [37, 38]; iii) Working Alliance Inventory – Short form Revised (WAI-SR) to assess therapeutic alliance between a patient and their clinician [39, 40]; and iv) Illness Perception Questionnaire Mental Health (IPQ-MH) to assess a patients’ perception of their illness with four of its sub-scales, namely, personal control over the problem, control gained in treatment, coherence, and emotional representation [41].

Baseline measurement (T0) includes i) a questionnaire on sociodemographic characteristics (gender, age, education level, work- and living situation), medication use, and past psychotherapy; ii) the Toronto Alexithymia Scale (TAS-20) to assess alexithymia (a subclinical inability to describe emotions), which may influence a participant’s ability to fill out the ESM diaries and thereby possibly influencing the efficacy of the Therap-i module [42–44]; iii) the Leiden Index of Depression Sensitivity - second revision (LEIDS-RR, [45, 46] to assess cognitive reactivity to negative mood to index depression vulnerability; iv) the Long-term difficulties questionnaire (e.g. housing or financial problems), which may partly mediate the severity of depressive symptomatology or interfere with the recovery process [47]; v) the Dysfunctional Attitude Scale (form A) revised (DAS-A-17 [48]; to assess the presence and intensity of dysfunctional attitudes; vi) the Childhood Trauma Questionnaire – Short Form (CTQ-SF) to assess the severity of childhood maltreatment, which may partly mediate the severity and/or chronicity of depressive symptomatology [49]. Some additional measurements are performed including i) the Questionnaire of Self-management in the Recovery from Depression (QSRD) at T4 to assess self-management using a descriptive outcome measure [50]. The QRSD originates from qualitative research in a sample of depressed patients, ii) the Brugha recent life events questionnaire, enriched with positive life events, at T4 to assess important positive and negative life events [51, 52], iii) the happiness index at T0 and T4, which is a single item, used in a large Dutch sample before, to assess a global estimation of how a participant feels in general [53, 54], and iv) information regarding TAU is collected, including medication switches (T3 and T7), applied types of psychotherapeutic treatment (T3 filled out by the clinician), and the number of sessions and treatment length in minutes (T7) collected from the electronic patient health record.

#### Actigraphy assessment

All participants wear an actigraph device (MotionWatch8®) for a period of 8 weeks. Actigraphy measurement starts together with ESM monitoring in the experimental group and is timed to start about 10 days after baseline in the control group to approximately match this timing (also see Fig. 1). Actigraphy data will be used for calculation of day-time activity patterns and circadian rhythms. Patients are instructed to push the button on the actigraph when going to sleep and right after waking up.

#### Qualitative evaluation interview

In the experimental arm only, participants and clinicians will evaluate the Therap-i module. The participants’ evaluation questionnaires include experienced utility of ESM (content, practicality, strain), feedback reports (content, intelligibility, lay-out), and feedback sessions (content, intelligibility). The clinicians’ evaluation questionnaires include the experienced utility of the Therap-i module supporting TAU. Both questionnaires are in-house instruments. Via a semi-structured interview, qualitative evaluations of the Therap-i module are obtained from 20 patients. The interview covers five topics: i) overall evaluation of the Therap-i module, ii) evaluation of the separate components of the Therap-i module, including the resilience interview, filling out the ESM diaries, feedback rapports, and the collaborative discussion based on the feedback rapports with the clinician and researcher iii) obtained insights by patients on their depression and if and how these insights were used in daily life to better manage depressive complaints, iv) influences of the Therap-i module on the psychological treatment, and v) influences of the Therap-i module on the therapeutic alliance.

### Data management

In the Therap-i study, the following types of data will be collected: questionnaire data, ESM data, logistic data, actigraphy data, interview data, personal data and work documents. Questionnaire and ESM-data are collected via the RoQua server [31]. RoQua is a secure web application which makes it possible to measure and monitor outcomes in health care and research. Logistic data will be collected via REDCap (Research Electronic Data Capture; https://www.project-redcap.org). REDCap is a secure web application for building and managing databases. Data collected in REDCap are automatically backed-up on a daily basis on the servers of the Universitair Medisch Centrum Groningen (UMCG). Logistic data concerns all data collected during the execution of research activities. For each procedural step, a digital questionnaire and checklist is built to support data capture and planning of research activities. Actigraphy data will be collected via CamNtech’s MotionWatch and downloaded via the software MotionWare. Interview data originate from the semi-structured interview as part of the qualitative evaluation of the Therap-i module. Personal data concern identifiable data, such as name, date of birth, (email) address, and telephone number. Work documents concern documents that are used in order to create the electronic diary or which contain feedback information discussed during a consult with the patient and therapist. All files are stored on a secured drive on the servers of the UMCG, which is accessible for authorized members of the research team only. Interviews are digitally recorded and the audio files will be transcribed verbatim. Work documents are scanned and stored as pdf-files. Paper versions are stored in a locked cabinet in our department. When the study closes, REDCap will be frozen after verification, all queries have been handled and the close-out visit of the monitor. Data files containing raw data are stored in a password protected folder on the secured drive. These data files as well as the actigraph, interview and personal data files will be locked for editing and contain the date of downloading/saving in their name. After publication of the main results of the study, the processed, pseudonymised data will be made available for re-use via a data transfer agreement.

### Statistical methods

#### Sample size calculation and withdrawal

We performed a sample size calculation for our main hypothesis that adding the Therap-i module to TAU will lead to greater reduction of depressive symptoms (as assessed with the IDS-SR) compared to TAU (G*Power 3.1, F test for repeated measures, within-between interactions [55]. Our power analysis is based on the effect size found in a previous study by Kramer and colleagues [21]. In their pioneer RCT, the authors showed that systematic intensive self-monitoring and personalized feedback on contextualized patterns of positive affect through ESM could provide an effective add-on tool to routine clinical care. IDS-SR scores for the experimental group showed an initial 3-point drop 7 weeks after baseline. While this effect became larger at later measurements, we chose the 7-week measurement as a reference point because it was the smallest post-intervention effect and therefore provided a conservative basis for the power analysis. Given a range of standard deviations of 10.0–10.5, the decrease in IDS-SR scores translates to an effect size of 0.14–0.15 (Cohen’s f). With a sample size of 80 (40 per group), an alpha of 0.05 and an intraclass correlation of 0.5 (between the pre and post repeated measures), and 4 measurement occasions (T0-T3), we have an 85–90% power to detect such an effect. To achieve a realistic estimation of the required sample size, one needs to further account for dropout. In a study from our department that is closely related to the proposed study [22, 23], dropout in the intervention period was 20%. Accordingly, to ensure that we will have at least 40 participants in each treatment arm, we will enrol 50 participants per study arm (total *n* = 100).

Patients can withdraw from the study at any time for any reason if they wish to do so without any consequences. In the case of withdrawal, attempts will be made to follow-up with the patient to establish cause of withdrawal, and to collect qualitative data regarding experience of participation. All data, including those from withdrawn patients (unless they request for their data to be deleted), will be included in the final analysis. If a patient withdraws from the study prematurely without providing valuable data, a replacement participant will be sought if resources allow. In case patients withdraw before randomization, they will be replaced. After randomization they will not be replaced.

#### Efficacy of the Therap-i module

The following analysis strategy will be applied to both primary (IDS-SR) and secondary outcomes (OQ-45, IPQ-MH subscales, and WAI-SR). The data have a two-level hierarchical structure, because multiple assessments are clustered within subjects. Therefore, multilevel regression analyses will be used to estimate fixed effects for time, treatment allocation, and their interaction, as well as random intercepts and a random slope for time. In this way, we can examine the effect of time on outcomes (across multiple outcome measurements), the effect of group (2 levels: experimental or control group), and the interaction between time and group separately. Thresholds for statistical significance will be set at *p* < 0.05.

#### Users evaluation of the Therap-i module

The evaluations of the Therap-i module by patients and clinicians will be assessed in two ways: quantitatively, and qualitatively through semi-structured interviews in a subsample of patients (*n* = 20). The quantitative data will be presented using descriptive statistics (means and standard deviations). The qualitative analyses will be performed according to the validated Qualitative Analysis Guide of Leuven (QUAGOL) procedure [56].

## Discussion

The ongoing global digitalisation influences the possibilities to monitor and manage our health and behaviours, and holds potential to improve outpatient mental healthcare. While routine outcome monitoring is being used to evaluate and compare treatment outcomes and process variables [57], the use of more frequent repeated measurements to assess psychopathological complaints has been limited. Currently existing monitoring routines are not fully taking advantage of the potential of these repeated measurements to obtain a fine-grained view on triggers and perpetuating factors of psychopathology. ESM offers the possibility to bring relevant information from outpatients’ daily life into regular consultations, enhancing a systematic personalized characterization of a patient’s psychopathology. In order to fulfil its promise to support precision psychiatry, ESM monitoring needs to be implemented in clinical practice, become part of the treatment process, and allow for personalization [58]. Until now, personalized ESM monitoring during psychotherapeutic treatment has not been researched as a supportive tool and with our pragmatic RCT study we aim to fill this gap. The unique contribution of our study is the integration of personalized ESM into regular outpatient treatment for depression. Quantitative and qualitative outcome variables will be combined to examine the added value of the Therap-i module. Specifically, the use of qualitative methods complements interests in clinical practice, since recovery and empowerment of patients are concepts which are difficult to grasp with quantitative measures. Data collection has started and will continue for at least another year. In short, the intent of the Therap-i module is to support the case conceptualisation process by increasing insight in a patient’s complaints and motivating change in behaviour through informed treatment strategies. As such the module may advance treatment efficacy for depression and the quality and duration of recovery from depression.

The following potential challenges should be mentioned. First, when interpreting our results, we have to take into account the selection bias of who was willing to enrol in the study. Not all patients and clinicians will be able, or are motivated, to invest time and effort needed to successfully participate. However, enrolment will show an accurate impression of the level of adoption of the module in this particular sample in clinical practice. Second, the study tests a combination of potentially active components in the Therap-i module: focus on resilience (via the resilience interview), ESM monitoring, the feedback rapport, and the collaborative discussion of the feedback results. Clear conclusions about the active ingredients are not warranted and more research will be needed for detailed insight into this matter. However, through our, quantitative and specifically qualitative evaluation of the Therap-i module, we gather information on these separate components to aid further testing and development. Third, the trial is “pragmatic” because it is designed to fit into regular clinical practice, which involves some flexibility in the procedure (e.g., different types and dose of TAU, different centers and therapists, a range of different comorbidities). The resulting heterogeneity may reduce statistical power and hide a potential effect of the Therap-i module. However, the fact that the trial is pragmatic also is a testament to the possibility to implement the module in clinical practice. Fourth, in case we find evidence that the Therap-i module is effective and a valuable contribution to the treatment of MDD patients, the findings cannot be generalized to other patient groups without additional study. Fifth, future large-scale clinical implementation of the Therap-i module would likely differ from this study because ideally patients and clinicians are able to implement the module independently without involvement of a researcher. To this end, therapists need to be trained and intuitive software needs to be developed.^1^

Despite these limitations, the Therap-i study will provide valuable insights into the promise of personalized ESM and ESM-based feedback in clinical practice. Currently, it is acknowledged that severe depressive problems are seldom fully cured as residual complaints often remain and that those affected have life-long vulnerabilities for relapse [59]. For this reason, the ultimate ambition behind our Therap-i module is to contribute to putting patients “back into the driver’s seat” and relieving them from the feeling that their depression is overpowering their lives.

## Supplementary Information

**Additional file: S1.** Fixed Experienced Sampling Methodology (ESM) items. **S2** Criteria for formulating ESM items.

## Data Availability

Data collection is ongoing therefore data and materials are not available. As such, there are currently neither publications containing the results of this study already published nor manuscripts in which results are described submitted to any journal.

## Abbreviations

CTQ-SF: Childhood Trauma Questionnaire Short Form
DAS-A-17: Dysfunctional Attitude Scale (form A) revised
DM-TRD: Dutch Method for quantification of Treatment Resistance in Depression
EMA: Ecological Momentary Assessment
ESM: Experienced Sampling Methodology
IDS-SR: Inventory of Depressive Symptomatology Self Report
IPQ-MH: Illness Perception Questionnaire Mental Health
LEIDS-RR: Leiden Index of Depression Sensitivity - second revision
MDD: Major Depressive Disorder
METc: Medical Ethical Committee
Mini-SCAN: Mini-Schedules for Clinical Assessment in Neuropsychology
OQ-45: Outcome Questionnaire
PETRA: PErsonalized Treatment by Real-time Assessment
QSRD: Questionnaire of Self-management in the Recovery from Depression
RCT: Randomized Controlled Trial
REDCap: Research Electronic Data Capture software
RoQua: Routine Outcome and QUality Assessment
TAS-20: Toronto Alexithymia Scale
TAU: Treatment As Usual
UMCG: University Medical Center Groningen
WAI-SR: Working Alliance Inventory – Short form Revised

## Footnotes

1 To meet this challenge, our group is currently developing the PErsonalized Treatment by Real-time Assessment (PETRA, petrapsy.nl) tool.
