# Supplementary material for: Personalized ESM monitoring and feedback to support psychological treatment for depression: a pragmatic randomized controlled trial (Therap-i)
Source: BMC Psychiatry. 2021 Mar 10;21:143. doi: 10.1186/s12888-021-03123-3 (PMC7945664; doi:10.1186/s12888-021-03123-3)
Supplement: Supplementary file 1 — Additional file: S1. Fixed Experienced Sampling Methodology (ESM) items. S2 Criteria for formulating ESM items. [file 12888_2021_3123_MOESM1_ESM.docx]

**Supplementary Materials**

**Personalized ESM monitoring and feedback to support psychological treatment for depression: A pragmatic randomized controlled trial (*Therap-i*)**

H. Riese, L. von Klipstein, R.A. Schoevers, D.C. van der Veen, M.N. Servaas

Table of Contents

[**S1 Fixed ESM items** 2](#_Toc62553448)

[**S2 Criteria for formulating ESM items** 5](#_Toc62553449)

[Criteria to be guarded by the researcher 5](#_Toc62553450)

[Criteria to discuss with participants 5](#_Toc62553451)

## **S1 Fixed ESM items**

Table S1.1. Fixed Experienced Sampling Methodology (ESM) items organized by domain (domain labels are in English [Dutch]). All items are assessed five times a day, except for the daily items that are assessed once a day (i.e. at the last beep of a day). Further details are given in the method section of the manuscript.

|  | English | Dutch | Response range | Range |
| --- | --- | --- | --- | --- |
| Feelings [gevoelens] | | |  |  |
| 1 | At this moment I feel satisfied | Op dit moment voel ik me tevreden | ‘not at all’ to ‘very much’ | 1-100 |
| 2 | At this moment I am cheerful | Op dit moment voel ik me opgewekt | ‘not at all’ to ‘very much’ | 1-100 |
| 3 | At this moment I feel down | Op dit moment voel ik me somber | ‘not at all’ to ‘very much’ | 1-100 |
| 4 | At this moment I feel irritated | Op dit moment voel ik me geïrriteerd | ‘not at all’ to ‘very much’ | 1-100 |
| 5 | At this moment I feel energetic | Op dit moment voel ik me energiek | ‘not at all’ to ‘very much’ | 1-100 |
| 6 | At this moment I feel restless | Op dit moment voel ik me onrustig | ‘not at all’ to ‘very much’ | 1-100 |
| Behavior [doen] | | |  |  |
| 7 | Since the previous beep, what have I done? | Sinds het vorige meetmoment, wat heb ik gedaan? | - work/study  - sports / walking / biking  - hobby (e.g., make music)  - outing (e.g., into the city, concert)  - something calm (e.g., reading, watching TV)  - sleep  - talk to somebody  - something intimate (e.g., cuddle, sex)  - messaging  - social media  - eat  - household responsibilities / groceries / administration  - self-care  - rest / nothing  - I was on the go  - something else | Checklist, multiple answers possible |
| Think [denken] | | | | |
| 8 | Since the previous beep, I have ruminated | Sinds het vorige meetmoment heb ik gepiekerd | ‘not at all’ to ‘very much’ | 1-100 |
| 9 | At this moment, my thoughts about myself are. | Op dit moment zijn mijn gedachten over mezelf | ‘very negative’ to ‘very positive’ | 1-100 |
| Body [lichaam] | | | | |
| 10 | At this moment, I am experiencing physical discomfort | Op dit moment ervaar ik lichamelijk ongemak | ‘not at all’ to ‘very much’ | 1-100 |
| 11 | Since the previous beep, I have been physically active | Sinds het vorige meetmoment ben ik lichamelijk actief geweest | ‘not at all’ to ‘very much’ | 1-100 |
| Context | | | | |
| 12 | At this moment, I am… | Op dit moment ben ik: | - alone  - in company  - alone with my pet | Checklist |
| a | *if in company:*  At this moment, with whom am I? | *indien in gezelschap:*  Op dit moment, met wie ben ik? | - partner  - housemates  - family  - family outside my household  - friends  - colleagues / classmates  - aid workers  - acquaintances  - strangers  - pet | Checklist, multiple answers possible |
| b | *if in company:*  I find this company pleasant | *indien in gezelschap:*  Ik vind dit gezelschap aangenaam | ‘not at all’ to ‘very much’ | 1-100 |
| c | *if alone:*  I would rather have been in company | *indien alleen:*  Ik zou liever in gezelschap zijn | ‘not at all’ to ‘very much’ | 1-100 |
| 13 | Think of the most pleasant event or activity since the last beep. How pleasant was this? | Denk aan de meest plezierige gebeurtenis of activiteit sinds het vorige meetmoment. Hoe plezierig was dit? | ‘not at all’ to ‘very much’ | 1-100 |
| 14 | How intense was it? | Hoe heftig was dit? | ‘not at all’ to ‘very much’ | 1-100 |
| 15 | Think of the most unpleasant event or activity since the last beep. How unpleasant was this? | Denk aan de meest onplezierige gebeurtenis of activiteit sinds het vorige meetmoment. Hoe onplezierig was dit? | ‘not at all’ to ‘very much’ | 1-100 |
| 16 | How intense was it? | Hoe heftig was dit? | ‘not at all’ to ‘very much’ | 1-100 |
| 17 | The (un-)pleasant experiences had to do with | De (on)plezierige ervaringen hadden te maken met | open string field | |
| 18 | If you want to note something else about the period since the last beep, you can do that here | Als u nog iets anders over de periode sinds het vorige meetmoment wilt noteren, dan kunt u dat hier doen | open string field | |
| Sleep | | | | |
| 19 | Is this the first measurement you fill out after the night? | Is dit de eerste meting die u invult na de nacht? | yes / no |  |
| a | *if yes:*  How was the quality of my sleep? | *zo ja:*  Hoe was de kwaliteit van mijn slaap? | ‘very bad’ to ‘very good’ | 1-100 |
| Daily items | | | | |
| 20 | Today I was able to enjoy things | Vandaag kon ik genieten | ‘not at all’ to ‘very much’ | 1-100 |
| 21 | Today I had a grip on how I was feeling | Vandaag ervoer ik grip op hoe het met me gaat | ‘not at all’ to ‘very much’ | 1-100 |
| 22 | Today I spend on things that I find important | Vandaag heb ik tijd besteed aan dingen die ik belangrijk vind | ‘not at all’ to ‘very much’ | 1-100 |

## **S2 Criteria for formulating ESM items**

### Criteria to be guarded by the researcher

1. Wording

1. Formulate as statement, not question
2. Do not create items with two opposite answering poles (e.g., negative to positive)
3. Use plain language, avoid research and psychology terms
4. Use straightforward language

2. Diminish affecting the participant

1. Items are about the ‘here and now’ and should not require much reflection (retrospective items are possible if justified by the content)
2. Avoid items that may lead to a strong emotional reaction in the participants

3. Clarity

1. Items should be about something that participants can think of themselves, that is independent of others
2. Items should be about one construct and avoid mixing multiple things together
3. Behavior that is observable should be prioritized

### Criteria to discuss with participants

1. Items should create enough variation. “Does this change over the course of the day?”
2. Items should cover aspects that are important. “Does this impact how you are doing? Is this an important indicator for how you are doing?”
